# Cancer-Associated Fibroblasts as the “Architect” of the Lung Cancer Immune Microenvironment: Multidimensional Roles and Synergistic Regulation with Radiotherapy

**DOI:** 10.3390/ijms26073234

**Published:** 2025-03-31

**Authors:** Zheng Shi, Cuilan Hu, Qiang Li, Chao Sun

**Affiliations:** 1 School of Biopharmaceutical and Engineering, Lanzhou Jiaotong University, Lanzhou 730070, China; 2 Institute of Modern Physics, Chinese Academy of Sciences, Lanzhou 730000, China; hucuilan@impcas.ac.cn (C.H.); liqiang@impcas.ac.cn (Q.L.); sunchao@impcas.ac.cn (C.S.); 3 University of Chinese Academy of Sciences, Beijing 101408, China

**Keywords:** cancer-associated fibroblasts, lung cancer, tumor immune microenvironment, radiotherapy

## Abstract

Cancer-associated fibroblasts (CAFs), as the “architect” of the immune microenvironment in lung cancer, play a multidimensional role in tumor progression and immune regulation. In this review, we summarize the heterogeneity of the origin and the molecular phenotype of CAFs in lung cancer, and explore the complex interactions between CAFs and multiple components of the tumor microenvironment, including the regulatory relationships with innate immune cells (e.g., tumor-associated macrophages, tumor-associated neutrophils), adaptive immune cells (e.g., T cells), and extracellular matrix (ECM). CAFs significantly influence tumor progression and immunomodulation through the secretion of cytokines, remodeling of the ECM, and the regulation of immune cell function significantly affects the immune escape and treatment resistance of tumors. In addition, this review also deeply explored the synergistic regulatory relationship between CAF and radiotherapy, revealing the key role of CAF in radiotherapy-induced remodeling of the immune microenvironment, which provides a new perspective for optimizing the comprehensive treatment strategy of lung cancer. By comprehensively analyzing the multidimensional roles of CAF and its interaction with radiotherapy, this review aims to provide a theoretical basis for the precise regulation of the immune microenvironment and clinical treatment of lung cancer.

## 1. Introduction

According to the latest statistics released by the International Agency for Research on Cancer (IARC) in 2022, lung cancer has emerged as the leading cause of both global cancer incidence and mortality [1]. Based on histological characteristics, lung cancer is categorized into two primary types: non-small cell lung cancer (NSCLC) and small cell lung cancer (SCLC). NSCLC is further classified into adenocarcinoma and squamous cell carcinoma, which represent its most common subtypes [2]. Radiotherapy is one of the very important treatment methods for lung cancer. A total of 77% of all patients with lung cancer have an evidence-based indication for radiotherapy at some point in their cancer journey [3]. In recent years, the rapid advancement of genetic testing and drug development has underscored the increasing significance of two precision treatment modalities in lung cancer therapy. The first is targeted therapy, which involves inhibitors like epidermal growth factor receptor (EGFR), activin receptor-like kinase (ALK), and reactive oxygen species (ROS)1 inhibitors designed to address driver gene mutations in lung cancer. The second is immunotherapy, where immune checkpoint inhibitors harness the body’s immune system to combat tumor cells, exemplified by PD-1/PD-L1 monoclonal antibodies [4]. Despite remarkable progress in targeted therapy and immunotherapy, treatment resistance continues to pose a significant challenge in the clinical management of lung cancer [5]. Recent studies have emphasized the pivotal role of the tumor microenvironment (TME) in driving resistance mechanisms [6]. The availability of immune checkpoint inhibitors (ICIs) targeting PD-1/PD-L1 or cytotoxic T-lymphocyte antigen 4 (CTLA-4) has revolutionized the treatment of NSCLC in the last decade. However, the chronic inflammatory environment present in lung cancer [7] may alter or deviate the differentiation of immune cells, leading to an imbalance in anti-tumor activity that favors tumor evasion [8], and subsequent resistance to ICIs [9]. Increasing evidence suggests that TME may be a promising biomarker of sensitivity to ICI based on the presence of immunomodulatory cells such as Treg, myeloid-derived suppressor cells, and tumor-associated macrophages. Therefore, several clinical studies have aimed at influencing TME towards a pro-immunogenic state, thereby increasing ICI activity [10]. For example, it was demonstrated that macrophage infiltration in NSCLC may be a determinant of PD1/PD-L1 antibody sensitivity and tolerance [11]. Additionally, vascular endothelial growth factor D (VEGFD) was found to be associated with innate immunity in patients with LUAD, which may serve as a potential predictor of LUAD patients receiving anti-PD-1 therapy [12]. Therefore, various possible regimens capable of optimizing the efficacy of ICIs are being progressively developed to meet clinical needs. It was demonstrated that an innovative small molecule inhibitor of PD-L1, S4-1, was able to induce PD-L1 internalization and optimize the immune microenvironment [13]. For targeted therapies, although Tyrosine Kinase Inhibitors (TKIs) are directly effective in killing NSCLC cells by inducing apoptosis, an increasing number of studies have demonstrated a close relationship between the regulation of the tumor immune microenvironment and the outcome of targeted therapies in lung cancer. For EGFR-TKI and ALK-TKI therapy, studies have demonstrated the dynamics of the TME during the use of different TKIs for the treatment of NSCLC, with an anti-tumor immune or immunostimulatory effect initially produced by the use of the TKIs, but an immunosuppressive effect after prolonged use which is the main reason for the emergence of resistance to the targeted therapy [14]. In the past decade, nanoparticle-based photoconductive therapy has shown great promise in the treatment of cancer, including lung cancer [15,16], with encouraging efficacy [17,18]. However, hypoxia affects the efficacy of phototransduction therapy, and a hypoxic tumor microenvironment is considered one of the hostile features of solid tumors [19]. VEGF is not only an important angiogenic factor, but also an immunomodulator of the TME. VEGF inhibits antigen presentation and stimulates the activity of regulatory T (Treg) cells and tumor-associated macrophages, which in turn promotes the formation of an immunosuppressive microenvironment in NSCLC. There is growing evidence that VEGF/VEGFR-targeted compounds and immunotherapy can serve as a new TME-targeted approach for the treatment of NSCLC [20]. Therefore, a comprehensive understanding of the TME’s components and its regulatory influence on tumor behavior will be essential for addressing the critical issue of treatment resistance in lung cancer.

In recent years, the TME has received increasing attention due to its critical role in tumor immunosuppression, distant metastasis, and targeted therapy response [21,22,23,24]. The tumor microenvironment is a highly complex system composed mainly of tumor cells, infiltrating immune cells (e.g., macrophages, dendritic cells, and lymphocytes), tumor-associated stromal cells (e.g., tumor-associated fibroblasts (CAFs), endothelial cells, and lipocytes, as well as the extracellular matrix (ECM) and multiple signaling molecules [25,26]. The tumor immune microenvironment (TIME) is mainly composed of different immune cell populations in the tumor, including immune cells and cytokines. These components are classified into anti-tumor components and pro-tumor components, and their interactions determine the trend of anti-tumor immunity, which is closely related to the immune status in the TME and the clinical prognosis of tumor patients [27,28,29]. CAFs are the most important component of TME, and activated CAFs can promote tumor growth, angiogenesis, invasion, and metastasis, as well as ECM remodeling through multiple pathways. The interaction between CAFs and TIME is considered to be another key factor in promoting tumor progression [28].

CAFs are heterogeneous and diverse in terms of morphology, function and their role in the tumor microenvironment. Their characteristics change dynamically in interaction with other cell types as the cancer progresses. This heterogeneity may affect tumor progression, treatment response, and prognosis [30]. The heterogeneity of CAFs and their multifaceted roles in lung cancer treatment are increasingly recognized as a critical area of research. CAF heterogeneity is closely linked to the TIME, particularly in mediating immune escape and immune suppression [31,32]. These cells influence the TME by secreting cytokines, remodeling the extracellular matrix, and modulating immune cell functions, thereby impacting the efficacy of both immunotherapy and radiotherapy. Emerging evidence suggests that CAFs contribute to the TIME not only by regulating immune cell infiltration but also through the expression of immune checkpoint molecules and the secretion of immunosuppressive factors, which collectively promote immune tolerance [33]. Radiotherapy, as an important therapy for cancer, not only directly targets tumor cells but also has the potential to modify CAF phenotypes or enhance their interactions with the immune system. This can amplify immune responses and synergize with immune checkpoint inhibition, ultimately counteracting immune suppression [34].

A comprehensive understanding of the intricate interplay between CAF heterogeneity and the immune microenvironment offers novel insights for refining combined strategies involving radiotherapy and immunotherapy.

## 2. Heterogeneity of CAF in Lung Cancer

### 2.1. Heterogeneity of CAF Origin

#### 2.1.1. Fibroblast

**Figure 1:**
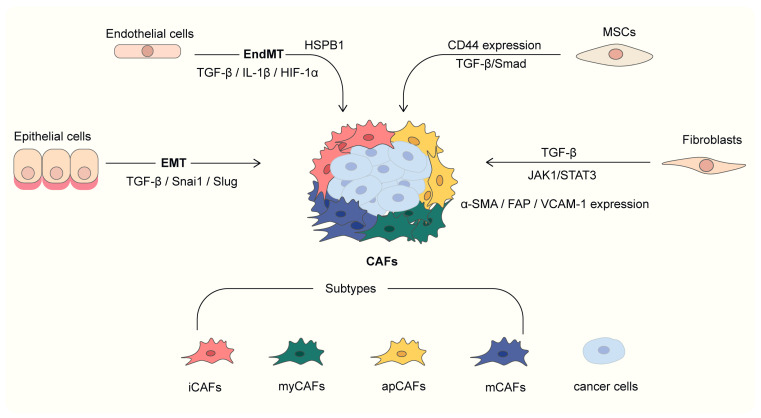
The heterogeneity of CAF origin and subtypes. Different colors represent different subtypes of CAFs.

As shown in Figure 1, the primary precursor of CAFs is normal fibroblasts, and their transdifferentiating into CAFs is predominantly driven by cancer-derived cytokines, such as transforming growth factor-β (TGF-β) [35]. This process is often accompanied by the remodeling of cellular phenotype and function, such as the upregulation of α-smooth muscle actin (α-SMA) and fibroblast activation protein (FAP) expression. Traditionally, this type of CAF has been regarded as a major driver of tumor stroma remodeling and tumor invasion [36]. Navab et al. found that TGF-β expression in CAFs is upregulated compared to normal fibroblasts [37]. LIF initiates an epigenetic switch that leads to constitutive activation of JAK1/STAT3 signaling, which results in sustained pro-invasive activity of CAF; inhibition of this pathway can chronically reverse CAF-associated pro-invasive activity and restore wild-type fibroblast phenotype [38]. Zhou et al. demonstrated that the expression of vascular cell adhesion molecule-1 (VCAM-1) in CAF-conditioned medium (CAF-CM) is higher than that in normal fibroblast-conditioned medium (NF-CM). VCAM-1 enhances the growth and invasion of lung cancer cells through the AKT and MAPK signaling pathways. Additionally, the analysis showed a positive correlation between the expression of α-SMA and VCAM-1, which is associated with poor prognosis in lung cancer patients [39]. Procopio et al. demonstrated that CSL silencing induces senescence in primary fibroblasts derived from various tissues, including the lung, supporting a model where CSL-p53 regulates CAF activation. Enhanced CAF activation simultaneously promotes the expansion of stromal cells and cancer cells [40].

#### 2.1.2. Epithelial-Mesenchymal Transition (EMT)

Epithelial cells can undergo transformation into CAFs through EMT, a process regulated by EMT-inducing factors such as transforming TGF-β, Snail, and Slug [41]. TGF-β, in particular, is the most potent inducer of EMT in NSCLC cells and is strongly associated with tumor invasion and metastasis [42]. Yang et al. isolated exosomes secreted by CAFs and normal fibroblasts and analyzed their effects on tumor volume and EMT. They demonstrated that miR-210 expression was significantly upregulated in CAF-derived exosomes (CAF-exo), which enhanced cell migration, proliferation, invasion, and EMT in NSCLC cells [43]. Similarly, You et al. showed that Snai1-dependent CAFs induce EMT in lung cancer cells through exosomes. The levels of Snai1 in CAF-derived exosomes correlated with Snai1 expression in CAFs, highlighting its critical role in promoting EMT in lung cancer cells [44]. Additionally, Wang et al. found that CAF-CM induces EMT by modulating the expression of EMT-related markers, such as E-cadherin and vimentin, and regulating metastasis-related genes, including MMP-2 and VEGF, both in vitro and in vivo [45]. In another study, Wang et al. demonstrated that CAFs isolated from lung adenocarcinoma enhance invasiveness and induce EMT by upregulating CXCR4, β-catenin, and PPARδ through the production of stromal cell-derived factor-1 (SDF-1) [46].

#### 2.1.3. Endothelial-Mesenchymal Transition (EndMT)

Endothelial cells can transform into CAFs through the process of EndMT. EndMT is driven by factors such as TGF-β, IL-1β, and HIF-1α and is closely associated with tumor angiogenesis and stromal remodeling [47]. Zeisberg et al. demonstrated that endothelial cells can undergo EndMT and transform into fibroblast-like cells. This transition is associated with the emergence of mesenchymal markers, including fibroblast-specific protein-1 (FSP1), and the downregulation of CD31/PECAM [48]. Ciszewski et al. found that TGF-β2 induces the mesenchymal transition of human microvascular endothelial cells (HMEC-1) into CAF-like cells, which is associated with the ILK-MMP9-MRTF axis. Higher levels of α-SMA protein and activation of the RhoA and Rac-1 pathways are critical for this differentiation. Overexpression of integrin-linked kinase (ILK) enhances myocardin-related transcription factor (MRTF) activation via the RhoA and Rac-1-MMP9 pathway, thus, promoting differentiation [49]. In addition, Choi et al. demonstrated that endothelial HSPB1 regulates EndMT in both pulmonary fibrosis and lung cancer. In clinical specimens of non-small cell lung cancer and lung tumor mouse models, HSPB1 plays an inhibitory role in EndMT [50].

#### 2.1.4. Bone Marrow-Derived Mesenchymal Stem Cells (BM-MSC)

BM-MSCs exhibit a propensity to migrate to tumor sites and differentiate into CAFs under the influence of tumor-derived factors, particularly TGF-β, thereby facilitating cancer progression [51]. These transformed CAFs are characterized by their robust secretion of pro-inflammatory cytokines and growth factors, which contribute to tumorigenesis. Notably, studies have demonstrated that bone marrow-derived macrophages can undergo transformation into CAF-like cells, thereby promoting pancreatic cancer progression, which underscores the remarkable plasticity of stromal cells within the TME [52]. The dynamic transdifferentiating of BM-MSCs into CAFs, influenced by wound fluid and tumor cell-secreted factors, further highlights the adaptive nature of stromal cell behavior in response to the TME [53]. Quante et al. provided compelling evidence that bone marrow-derived myofibroblasts not only contribute to the MSC niche but also actively promote tumor growth, suggesting a potential mechanistic link between MSCs and cancer progression [54]. Furthermore, Spaeth et al. demonstrated that CD44 expression in MSCs plays a crucial role in the activation of the fibroblast phenotype within the tumor microenvironment. Specifically, the attenuation of CD44 in MSCs was shown to significantly limit their expression of tumor-regulated CAF markers [55]. In a related study, Peng et al. revealed that tumor-derived glucose-regulated protein 78 (GRP78) treatment induces the expression of the CAF marker α-SMA in both human bone marrow mesenchymal stem cells (HBMSCs) and mouse bone marrow mesenchymal stem cells (BMMSCs). This differentiation of BMSCs into CAFs is mediated through the activation of the TGF-β/Smad signaling pathway [56].

#### 2.1.5. Methods for Separating CAFs from TME

Isolation of fibroblasts from tumor samples is a crucial step for understanding the tumor microenvironment, their interactions with immune cells, and their impact on cancer progression. Various studies have focused on distinct methodologies for isolating fibroblasts from tumor specimens. Sha et al. employed a trypsin-collagenase I combined digestion method to process tumor tissues and non-tumorigenic tissue samples from cancer patients. After filtration through a 70 μm membrane, CAFs and normal fibroblasts were selected using anti-fibroblast microbead MACS technology [57]. Zawieracz et al. de-scribed methods for isolating primary cancer-associated fibroblasts and fibroblasts from omental tissue using mechanical dissociation and enzymatic digestion [58]. Sharon et al. developed a strategy based on the surface marker PDGFRα to isolate purified normal fibroblasts and CAFs from fresh mouse and human tissues (including breast, pancreas, lung, etc.) via fluorescence-activated cell sorting. This approach overcomes gene expression bias caused by in vitro culture and enables unbiased transcriptional analysis of heterogeneous CAF subpopulations. It also supports subsequent functional studies free from tumor contamination [59]. Jiang et al. introduced a rapid isolation method for circulating cancer-associated fibroblasts (cCAFs) from blood samples of cancer patients. The authors developed a label-free, high-throughput microfluidic technique based on microbubble acoustics, capable of separating circulating tumor cells, immune cells (e.g., leukocytes), and rare circulating populations such as cCAFs from clinically diagnosed patients [60].

### 2.2. Heterogeneity of Molecular Phenotype

#### 2.2.1. Classical Molecular Markers of CAFs

Based on the available studies, the common markers of CAFs in lung cancer are summarized in Table 1. Many of the markers in the table are also suitable for the identification of CAFs in other tumor types [61,62]. Among them, the more important ones are FAP, α-SMA, and PDGFRβ, which will be specifically described below.

1. FAP

FAP is a cell surface glycoprotein predominantly expressed on activated fibroblasts within tumor tissues, particularly in CAFs. As a type II transmembrane serine protease, FAP exhibits both collagenase and gelatinase activities, playing a pivotal role in ECM remodeling and facilitating cancer cell migration, invasion, and metastasis [72]. Elevated FAP expression has been observed in various cancers, including lung cancer, and is correlated with poor prognosis across multiple cancer types. Wang et al. investigated the metastatic potential of lung cancer cells influenced by CAFs, demonstrating that stromal fibroblasts isolated from lung cancer tissues displayed CAF characteristics, marked by high expression levels of α-SMA and FAP [45]. Kilvaer et al. explored the potential immune-adjuvant role of FAP-1-positive fibroblasts in NSCLC, suggesting a link between CAFs and immune markers within the tumor stroma [73]. Chen et al. examined the clinical significance of FAP-α in lung squamous cell carcinoma, revealing that higher CAF density and lymph node metastasis were negatively correlated with patient survival [74]. Fang et al. developed FAP-targeted chimeric antigen receptor NK-92 cells for NSCLC, demonstrating increased FAP expression in A549+CAF and H226+CAF cells in nude mouse models [75]. Mathieson et al. identified that specific CAF subpopulations expressing FAP and podoplanin in NSCLC serve as predictors of poor clinical outcomes, highlighting the importance of characterizing CAF subtypes in NSCLC [64]. Peltier et al. discovered that FAP is detectable on the surface of CAFs in numerous cancer types associated with poor prognosis. Consequently, they developed and validated 68Ga-labeled radiopharmaceutical FAP inhibitors (FAPIs) for whole-body imaging of FAP expression using positron emission tomography (PET/CT) [63].

2. α-SMA

Wang et al. investigated the influence of CAFs on the metastatic potential of lung cancer cells, revealing that fibroblasts isolated from lung cancer tissue exhibited CAF characteristics, characterized by elevated levels of α-SMA [45]. Li et al. demonstrated that CAF markers, including α-SMA, vimentin, and CAF-specific factors such as CXC motif chemokine ligand 2 (CXCL12), FGF10, interleukin-6 (IL-6), and collagen type I alpha 1 chain (COL1A1), were highly expressed in senescent fibroblasts, which contributed to the promotion of lung cancer cell migration and invasion. These senescent fibroblasts acquired a CAF phenotype through a Stat3-dependent mechanism [76]. Additionally, Zhou et al. highlighted the role of VCAM-1 secreted by CAFs in enhancing lung cancer cell growth and invasion via the AKT and MAPK signaling pathways. Their studies revealed a positive correlation between the expression of CAF marker proteins, α-SMA and VCAM-1, which was associated with poor prognosis in lung cancer patients. These findings underscore the critical role of CAFs in driving lung cancer invasiveness [39]. In contrast, Lu et al. focused on SCLC and explored the dynamic phenotypic reprogramming induced by lung fibroblasts in SCLC. They identified a positive correlation between the expression levels of α-SMA, CAF markers, and REST (a protein typically expressed in non-neuroendocrine SCLC. This study emphasizes the importance of understanding the interplay between CAFs and non-neuroendocrine tumor cells within the tumor microenvironment [77].

3. PDGFR-α/β

Platelet-derived growth factor receptor (PDGFR) is a receptor tyrosine kinase that plays a pivotal role in modulating the behavior of fibroblasts and other cellular components within the tumor microenvironment. PDGFR-α and PDGFR-β have been recognized as key markers for fibroblast identification and are frequently upregulated in various malignancies, including lung cancer [78]. Both the PDGFR-α homodimer and the PDGFR-α/β heterodimer are implicated in the functional activities of CAFs. Emerging evidence suggests that matrix reprogramming through dual PDGFRα/β inhibition holds potential for enhancing therapeutic outcomes in gastric and lung cancers [79]. Transgelin, a protein implicated in promoting lung cancer progression, has been linked to elevated levels of α-SMA and PDGFR-β, further underscoring the significance of these markers in the functional regulation of CAFs [80]. Additionally, Sugai et al. assessed the expression of CAF-related proteins, including PDGFR-α and PDGFR-β, in lung squamous cell carcinoma (LSCC) tissues using immunohistochemistry to evaluate their impact on clinical outcomes [64].

#### 2.2.2. Subtypes of CAF Based on Molecular Phenotypes

1. MyCAF

The heterogeneity of CAF subtypes is also briefly represented in Figure 1. Myofibroblast-like cancer-associated fibroblasts (myCAFs) represent a distinct subset of CAFs within the TME, prominently identified in malignancies such as pancreatic ductal adenocarcinoma (PDAC) and head and neck squamous cell carcinoma (HNSCC). Research has demonstrated that integrin α11β1 is expressed at moderate levels in myCAFs, while it is notably absent in other stromal cell types, including FSP1-positive and neuron-glial antigen 2 (NG2)-positive cells [81]. In lung adenocarcinoma (LUAD), myCAFs have been identified as the major subtype of CAFs [82]. myCAF is rich in ECM transcripts and can enhance the invasion and metastatic ability of lung cancer cells by remodeling the ECM and secreting chemokines. The secreted matrix metalloproteinases (MMPs) play a key role in tumor invasion and metastasis by promoting cell migration and ECM degradation [83].

2. iCAF

Inflammatory CAFs (iCAFs) are also one of the more predominant subtypes of CAFs [84,85]. Xu et al. identified three distinct CAF subpopulations in NSCLC using single-cell sequencing. Their findings suggest that iCAFs may interact with NSCLC cells by activating molecules associated with invasion and metastasis, such as MET-hepatocyte growth factor (HGF) signaling pathway, thereby promoting brain metastasis in NSCLC [86]. In a spatially resolved single-cell imaging mass cytometry (IMC) analysis of CAFs from 1070 NSCLC patients, heterogeneity of CAFs was found to be an independent prognostic factor affecting patient survival. In particular, iCAF was associated with an inflammatory tumor microenvironment and higher patient survival [87]. Cords et al. discovered that the CAF phenotype is an independent prognostic factor for NSCLC, with iCAFs and interferon response CAFs being associated with an inflamed TME and improved patient survival [87]. Additionally, Zhao et al. revealed that the circRNA-mediated fibroblast niche plays a critical role in tumor metastasis. Specifically, the circNOX4/miR-329-5p/FAP axis activates the iCAFs niche by preferentially inducing IL-6, ultimately driving NSCLC progression [88].

3. Other subtypes

Kim et al. identified a new subgroup of CAFs in lung adenocarcinoma, antigen-presenting CAFs (apCAF) via RAN-seq, and demonstrated that some of their markers, UBE2T and KPNA2, promote CAF aggressiveness [64]. Lu et al. demonstrated that crosstalk between CAFs and non-neuroendocrine (non-NE) SCLC cells promoted the presence of apCAFs, which may contribute to CD8^+^ T cell capture and Treg differentiation [89]. Chen et al. identified matCAF, suggesting that matCAF plays a crucial role in cancer development and progression [84]. In studies for NSCLC, it has been demonstrated that matrix CAF (mCAF) was associated with low immune infiltration and low patient survival [87].

#### 2.2.3. Subtypes of CAFs in Different Lung Cancer Types

In NSCLC, CAFs exhibit significant heterogeneity, encompassing distinct subtypes such as myCAF, iCAF, apCAF, and matCAF [90]. Notably, CAFs with myofibroblast characteristics have been identified as a potential marker of malignancy in advanced NSCLC [91]. Hu et al. established an in vivo CAF biobank and identified three major functional CAF subtypes: Subtype I, which overcomes EGFR inhibitor resistance via the MET pathway and highly expresses HGF and FGF7; Subtype II, which mediates moderate resistance primarily through the FGFR pathway and also expresses FGF7; and Subtype III, which exhibits a weaker protective effect. These subtypes are regulated by the TGF-β pathway, and their functional differences significantly influence therapeutic responses to EGFR and ALK TKIs [92]. Hanley et al. utilized single-cell transcriptomic analysis to reveal an enriched myoCAF gene signature in NSCLC compared to normal lung fibroblasts, while iCAF-associated genes were more prominent in normal lung tissues [93].

In LUAD, one study identified five CAF subgroups in LUAD, three of which were associated with prognosis. Based on the prognostic significance of CAF subgroups, the study constructed a risk characterization model containing eight genes, including one protective gene (BTK) and three risk genes (CCNB1, CDC25C and EXO1). The CAF-based risk profile model can effectively predict the prognosis of LUAD patients and provide a new strategy for interpreting the response of LUAD to immunotherapy [94]. Another study found that Thy-1^+^ CAF (tCAF) localized in metastatic foci and formed tCAF-dominated invasive structures under ZEB1-driven REPULSIVE FORCES, supporting the notion that CAF facilitates invasion through mechanical forces [95].

In LUSC, single-cell RNA sequencing classified diverse CAF subtypes. Univariate Cox analysis identified prognostic CAF-related genes (CAFRGs), revealing correlations between CAFRGs and malignant features such as tumor angiogenesis, EMT, and cell cycle dysregulation [96]. Fang et al. understood the heterogeneity of the tumor microenvironment in lung adenocarcinoma and squamous carcinoma using single-cell transcriptomic analysis and found that infiltrating CAF were elevated in LUSC and up-regulated immunosuppressive extracellular matrix remodeling [97]. Notably, Ki67 exhibits divergent prognostic roles in LUAD and LUSC, reflecting tumor proliferation heterogeneity. High Ki67 expression predicts poor prognosis in LUAD, whereas low Ki67 levels correlate with elevated EMT potential and worse outcomes in LUSC [98].

SCLC is a highly malignant lung cancer type in which the role of CAF in TME remains incompletely defined. However, it has been shown that the non-neuroendocrine (non-NE) subtype of SCLC may be associated with the presence of apCAF. CAF regulates the immune microenvironment of the tumor through CD8^+^ T-cell capture and Treg differentiation, which in turn affects the therapeutic response of SCLC [89].

## 3. CAF and Tumor Immune Microenvironment in Lung Cancer

**Figure 2:**
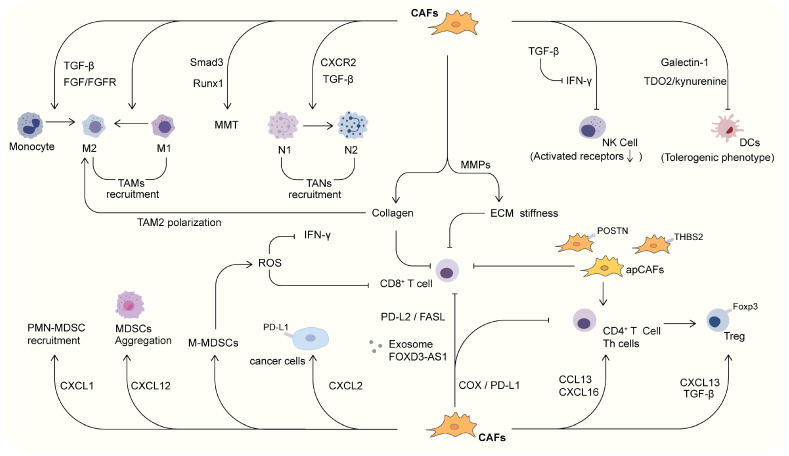
The relationships between CAF and tumor immune microenvironment in lung cancer.

Much evidence has demonstrated that CAFs play an important role in the regulation of TIME. CAFs are able to regulate the antitumor activity of tumor-infiltrating immune cells [99], and are able to promote the regulation of ECM remodeling and immune checkpoint molecules, which indirectly affects the activity and recruitment of immune cells [100]. In addition, CAF are involved in cancer development and progression through the secretion of chemokines, cytokines, and other effector molecules, including TGF-β, CXCL2, collagen, MMPs, and laminin [101,102]. One study found that in NSCLC, the FBLIM1-positive CAF subtype is an aggressive subtype associated with increased TGF-β expression, elevated levels of mesenchymal markers and an immunosuppressive tumor microenvironment [103]. In a study of CAFs in NSCLC patients, CAFs with high expression of both HGF and FGF7, and CAFs with high expression of FGF7 were found to be differentially protective against tumors, and were associated with the immune microenvironment of tumors [92]. In short, CAF is closely related to the immune microenvironment of lung cancer, and can influence the recruitment and function of multiple immune cells, which leads to the regulation of the immune status. The relationships between CAF and innate immune cells, adaptive immune cells, and ECM are discussed separately below and summarized in Figure 2.

### 3.1. Connection Between CAF and Innate Immune Cells

#### 3.1.1. CAF and TAMs

Tumor-infiltrating macrophages are known as tumor-associated macrophages (TAMs) and can be divided into two subtypes, M1 and M2 [104]. M1-type macrophages exhibit antitumor effects in TIME mainly by mediating the production of ROS, cytotoxicity, and tumor necrosis factor (TNF) [105], whereas M2 macrophages exhibit pro-tumor activity by activating cancer cell invasion and metastasis, tumor angiogenesis, immunosuppression, and ECM remodeling [106,107]. TAMs are the most prominent immune cells in proximity to CAFs, and there is a close interaction between the two cells [108]. It has been shown that CAFs can promote the recruitment of monocytes (precursors of macrophages) and differentiate them into M2-type TAMs through a variety of regulatory molecules, resulting in immunosuppression [109]. The same trend has been found in prostate cancer as well as breast cancer [110,111]. CAFs in lung adenocarcinoma promoting the polarization of TAMs to the M2 phenotype are associated with the FGF/FGFR signaling pathway [112]. For CAF subtypes, it has been found that PDPN^+^ CAFs exhibit higher TGF-β expression and are associated with the infiltration of CD204^+^ TAMs (M2 phenotype TAMs) in lung cancer, which in turn contributes to an immunosuppressive TME [113]. POSTN^+^ CAFs can bind to SPP1^+^ TAMs, which in turn contribute to the immunosuppression of NSCLC [114]. Furthermore, macrophage-myofibroblast transformation (MMT) is known to exist in lung cancer to support cancer progression, and this transformation may be Smad3-dependent [115]. The hematopoietic transcription factor Runx1, a key regulator downstream of Smad3 signaling, is positively associated with MMT-derived CAF abundance and mortality in NSCLC patients [116].

#### 3.1.2. CAF and TANs

Tumor-associated neutrophils (TANs) are also one of the important constituents of TIME. Tumor-produced chemokines attract neutrophils in the blood circulation, which pass through the vessel wall into the tumor tissue and then form TANs. Once TANs are activated in the tumor microenvironment, they appear to increase the complexity of the inflammatory environment through a mechanism of attraction to other leukocytes [117]. TANs play an important role in many aspects of tumorigenesis and development, such as malignant transformation, tumor progression, extracellular matrix modification, angiogenesis, cell migration, and immunosuppression [118,119,120]. Similar to TAM, it has been shown that neutrophils can be categorized into N1 and N2 types, representing tumor suppressor and tumor promoter phenotypes, respectively [121]. Studies have demonstrated that CAFs promote the polarization of N2 TANs in lung cancer [122]. C-X-C chemokine receptor 2 (CXCR2), a cytokine receptor expressed by CAFs, has been shown to be a major factor involved in tumor neutrophil recruitment and migration [121,123]. It has been proved in lung cancer as well as in mesothelioma that TGF-β plays an important role in the polarization of the TANs phenotype [124]). Additionally, the transdifferentiating of fibroblasts to CAFs is largely driven by TGF-β [35,125]. Furthermore, studies on lung cancer have found that granulocyte colony-stimulating factor (G-CSF) and granulocyte-macrophage colony-stimulating factor (GM-CSF) induce immune escape from lung cancer by regulating neutrophils [126].

#### 3.1.3. CAF and NK Cells

Natural killer (NK) cells are members of the innate immune system that respond to tumor cells with a variety of effector functions, primarily cell killing and the production of pro-inflammatory cytokines [127,128,129] The cytotoxic activity of NK cells makes them most functionally similar to CD8^+^ T cells [129]. The activity of NK cells is dependent on the expression and stimulation of activating or inhibitory receptors on the cell surface [130,131]. A growing number of studies have shown that CAFs are able to exert effects on NK cells by direct or indirect means [132,133]. In lung cancer, CAFs are able to exert significant immunosuppressive effects on NK cells [134]. TGF-β is recognized as a key cytokine connecting CAFs and NK cells in tumors [135]. TGF-β secreted by CAFs significantly inhibits the activation and cytotoxic activity of NK cells [136]. The mechanism may be due to the fact that TGF-β reduces the production of interferon-γ (IFN-γ) and down-regulates activated receptors on the cell surface [137].

#### 3.1.4. CAF and DCs

Tumor-infiltrating dendritic cells (DCs) are initiators and coordinators of the adaptive immune response and are essential for the anti-tumor immune response [138]. DCs are essential for T-cell-mediated cancer immunity; they transport tumor antigens to the draining lymph nodes and cross-present antigens to activate cytotoxic T lymphocytes [139]. In recent years, several studies have shown that CAFs can lead to the immune escape of tumor cells by blocking processes such as DC maturation and antigen presentation. In lung cancer, CAFs can induce a tolerogenic phenotype in DCs [140]. Galectin-1 derived from lung cancer is one of the culprits of DC cell incapacitation [141], and it has been demonstrated that this phenomenon is mediated by the galectin-1-CAF axis and mainly associated with the tryptophan 2,3-dioxygenase (TDO2)/kynurenine axis in CAFs [142]. Additionally, researchers have found that immunomodulation through inhibition of CAF function improves regional and systemic antitumor immune responses, thereby increasing the efficacy of DC-based vaccine immunotherapy [143].

#### 3.1.5. CAF and MDSCs

Myeloid-derived suppressor cells (MDSCs) originate from the bone marrow and have potent immunosuppressive activity in TIME [144]. MDSCs are a heterogeneous population of myeloid cells, which are a mixture of myeloid cells with the phenotype of granulocytes and monocytes, but lack the expression of cell surface markers specific for monocytes, macrophages, or DC, and are effective in suppressing both innate and adaptive immune responses, especially in T cell proliferation and cytokine production [145]. In LSCC, CAF is able to induce the aggregation of mononuclear MDSCs (M-MDSCs) and the generation of large amounts of ROS, which ultimately leads to the inhibition of CD8+ T cell growth, as well as the restricted production of IFN-γ, resulting in immunosuppression [146]. Fibrinogen-like protein 2 (Fgl2), a pleiotropic cytokine affecting multiple cellular functions, was found to be able to increase the aggregation of MDSCs through CXCL12 in lung cancer, and also induced the activation and pro-tumorigenic phenotype of CAFs, which are the main source of CXCL12 in TME [147]. Moreover, CAFs produce the granulocyte chemokine CXCL1, which may also be involved in the recruitment of polymorphonuclear myeloid-derived suppressor cells (PMN-MDSC) [148].

### 3.2. Connection Between CAF and Adaptive Immune Cells

T lymphocytes play a key role in regulating the adaptive immune response, and they consist of different subpopulations such as Treg cells, helper T (Th) cells, and cytotoxic T lymphocytes (CTL) [149]. The success of immune checkpoint therapy suggests that tumor-reactive T cells can destroy cancer cells but are constrained by immunosuppression in the TME. CAF are the major stromal cells in the TME and co-localize with T cells in lung cancer [150].

#### 3.2.1. CAF and Treg Cells

Forkhead box P3 (Foxp3) highly expressed Treg cells have an important function in limiting anti-tumor immunity [151]. In lung cancer, it was demonstrated that Treg cells were adjacent to CAFs and that infiltration of Foxp3^+^ Tregs and CAFs in the tumor stroma correlated with poor prognosis [152]. Moreover, CAFs expressing immunomodulatory cytokines may induce Tregs in the stroma, resulting in a tumor-promoting microenvironment in lung adenocarcinoma that leads to a poor prognosis [152]. CAFs in NSCLC amplify a Treg phenotype in TME by driving the production of CXCL13 from activated T cells via TGF-β [153]. In a recent study targeting SCLC, apCAFs were shown to promote glycolysis in tumor cells and induce the formation of Tregs from naive CD4^+^ T cells in an antigen-specific manner [89].

#### 3.2.2. CAF and Th Cells

The cell subsets mainly include Th1, Th2, and Th17 cells, which mostly differentiate from naive CD4^+^ T cells [154]. Th1 and Th2 cells are involved in cellular and humoral immunity, respectively, through the secretion of a variety of specific cytokines [155]. The presence of CAFs induced infiltration of B cells and CD69^+^CD4^+^ T cells, which was associated with increased expression of CCL13 and increased expression of CXCL16 [156]. Meflin-positive CAF enhanced the tumor response to immune checkpoint blockade (ICB) therapy, positively correlating with CD4^+^ T-cell infiltration and vascularization [157].

#### 3.2.3. CAF and CTLs

CD8^+^ T cells, also known as CTLs, mediate cytotoxicity mainly by inducing apoptosis in tumor cells, which is the most critical component of anti-tumor immunity [155]. Numerous studies have reported interactions between CAFs and CD8^+^ T cells in lung cancer [158]. THBS2^+^ CAFs are capable of interacting with CD8^+^ T cells and are associated with immunosuppression in lung adenocarcinoma [159]. It was found that apCAFs may capture CD8^+^ T cells and trap them in the stromal region [89]. POSTN^+^ CAFs are able to bind to SPP1^+^ macrophages and cause inhibition of T-cell infiltration, and have been associated with a poor prognosis in NSCLC [114]. The CD8/Foxp3+ T-cell ratio tends to be lower in the region of PDPN^+^ CAFs, resulting in a suppressive immune microenvironment [160]. Two types of CAFs, MYH11^+^αSMA^+^ CAF and FAP^+^αSMA^+^ CAF, were also found to be associated with T-cell marginalization [161]. Hyewon et al. found that CAFs inhibit the function of CD4^+^ and CD8^+^ T-cells in NSCLC through their effects on COX2 and PD-L1, thereby promoting immune escape [162]. Another study demonstrated that CAF significantly increased CXCL13 production by CD4^+^ and CD8^+^ T cells via TGF-β, thereby altering tumor TIME [153]. Mao et al. demonstrated that the CD8^+^ T cell-derived exosome miR-2682 inhibited lung cancer tumor formation, whereas the CAF-derived exosome FOXD3-AS1 promoted lung cancer tumor formation [163]. In addition to this, the CD8^+^ T cell/CAF ratio (CFR) allows for prognostic stratification as well as response prediction to immunotherapy in multiple cancer types [164]. In addition to the regulatory effect of CAF on T cells, studies have demonstrated that there is also a negative feedback mechanism of T cells on CAF, promoting the expression of co-suppressor ligands, CD73 and IL-27 in NSCLC [150]. A recent study indicated that T-cell effector cytokines IFN-γ and TNF-α induced cytokine and chemokine secretion from NSCLC patient-derived CAFs in vitro, which may affect immune cell recruitment and activation and, ultimately, tumor progression and treatment response [165].

### 3.3. Interactions Between CAF and Other Factors in TIME

High expression of immune checkpoint molecules on the surface of T cells and tumor cells has been identified as a major cause of immunosuppression of T lymphocytes [166,167]. PD-L1 and PD-1 are well-known checkpoint molecules. The binding of PD-L1 to PD-1, a receptor on activated T cells, counteracts T cell activation signals and, thus, prevents anti-tumor immune response [168]. It was found that in lung cancer, CAF was able to inhibit T cells by activating immune checkpoints via PD-L2 and FASL [169]. Similarly, researchers found that CAF expressing PD-L1 and PD-L2 in NSCLC were affected by IFN-γ and influenced T cell function [170]. FAP was found to predict the efficacy of PD-1 blockade therapy in advanced non-small cell lung cancer [171]. Furthermore, WDR62 was significantly up-regulated in most tumors, and WDR62 expression was associated with the infiltration of a variety of immune cells, particularly CAF and Treg cells, and was significantly correlated with poor prognosis. Studies have shown that WDR62 predicts the efficacy of immunotherapy, particularly PD1/PD-L1 inhibitors [172]. In addition to upregulating molecules on their own surface, CAFs produce various cytokines and exosomes, which indirectly exert an inhibitory effect on T cell function and antitumor responses. By secreting factors such as CXCL2, CAFs can increase the expression of PD-L1 in lung adenocarcinoma cells, thereby affecting tumor immunity [173].

ECM is a complex network of different macromolecules such as collagen, fibronectin, glycoproteins, and proteoglycans, which are responsible for maintaining the structure, integrity, and homeostasis of normal tissues [174,175]. By secreting a variety of matrix proteins and producing a number of matrix metalloproteinases (MMPs), CAFs promote the degradation of normal ECM structures and increase matrix stiffness [175,176]. Dysregulation of the ECM has been found to be associated with activation of TGF-β signaling in CAFs and with immunosuppression of tumors [177]. This remodeled ECM acts as a physical barrier for immune cells, thereby inhibiting their recruitment to the cancer site and reducing the immune response [178,179]. The density of collagen in lung cancer has been found to influence anti-tumor immunity, and in areas of dense stroma, the ability of T cells to reach the cancer site is limited [180]. In addition, ECM remodeled by CAF modulates the activity and function of other immune cell populations. CAF-induced collagen-rich stroma is associated with TAM recruitment and function. In lung cancer, this aberrant collagen-rich ECM induces M2-type polarization of macrophages, and collagen monomer-induced innate immune responses may perpetuate lung fibrosis [181]. COL11A1^+^ CAF promotes the progression of lung adenocarcinoma and bladder cancer by regulating ECM remodeling and anti-tumor immune responses [182]. Conversely, it has been found that in lung adenocarcinoma, ADH1B^+^ CAF is associated with better survival. Reduced COL1A1 and COL3A1 expression and increased DCN, LAMA2, and ELN expression have been characterized in ADH1B^+^ CAF, and correlate with the function of the ECM [82].

In conclusion, the heterogeneity of CAF is capable of exerting diverse effects on immune checkpoint molecules and the ECM, and generating different responses to anti-tumor immune responses.

## 4. Relationship Between CAF and Radiotherapy

The treatment of lung cancer is complex, and radiotherapy (RT) can be used in all stages of the disease for curative or palliative care. With advances in technology, radiotherapy allows for better targeting of the tumor and reduces incidental irradiation of surrounding normal tissue. This has expanded the indications for radiotherapy in lung cancer and improved outcomes in terms of increased survival and reduced toxicity [3]. However, RT has been shown to be a double-edged sword that not only enhances systemic anti-tumor immune responses, but also leads to immunosuppression [183]. For example, radiotherapy promotes anti-tumor immunity, activates cytotoxic T cells, and enhances the killing effect on tumor cells [184]. In addition, radiotherapy induces the upregulation of immunosuppressive molecules, promotes the inactivation of immune effector cells, weakens the immunogenicity of tumor cells, and ultimately leads to the development of immune escape and tolerance [185]. Therefore, the alteration of TME by RT affects the anti-tumor immune response in multiple ways.

Many studies have shown that radiation can alter TME by affecting the phenotype of CAFs. For example, in NSCLC, the upregulation of some immune receptors such as CD73 and CD276 on the surface of irradiated CAFs contributes to the formation of an immunosuppressed TME after radiotherapy [183]. Radiotherapy-induced generation of senescence-like CAFs promotes the proliferation and radioresistance of NSCLC cells through the JAK/STAT pathway. Specific induction of senescence-like CAF apoptosis using the FOXO4-p53 interfering peptide FOXO4-DRI reduces both NSCLC radioresistance and incidence of radiation-induced pulmonary fibrosis (RIPF) [186]. Irradiation-induced elevation of immune receptors on the cell surface of CAFs may contribute to the establishment of immunosuppressive TME after radiotherapy [183]. It has been found that although much evidence suggests that high-dose (>5 Gy) radiotherapy regimens have a stronger pro-immunogenic effect than standard low-dose (2 Gy) regimens, CAFs have a potent immunosuppressive effect on activated T-cells, and this effect is maintained after high-dose radiotherapy (HD-RT). Importantly, CAFs do not initiate an immunogenic cell death response after exposure to HD-RT [187]. CAFs in irradiated NSCLC were able to maintain immunosuppressive effects on NK cells [134]. One study selected 68 patients with brain metastases from different primary cancer types, including 10 lung cancer patients, and evaluated the expression and clinical relevance of CAF-related biomarkers in brain metastases (BM), and showed that PDGFR-β and α-SMA were highly expressed in patients who had received chemotherapy or radiotherapy for their primary cancers, and that they were associated with a poor prognosis and recurrence in BM patients [188]. In another study, CAFs were isolated from lung tumor specimens of 16 NSCLC patients, and the results showed that radiation caused a sustained enhancement of the surface expression of integrins α2, β1 and α5, and that radiation had a favorable inhibitory effect on the proliferative, migratory and invasive capacities of CAFs in NSCLC [189]. However, conversely, it has also been shown that integrin β1 allows SCLC cells to survive in the presence of sustained DNA damage [190]. The fibroblast integrin α11β1 has been shown to induce myofibroblast activation [191]. Integrin αVβ6 has been reported to be upregulated prior to the onset of fibrosis in irradiated lungs, suggesting that integrins may also be involved in the development of radiation fibrosis [192]. In addition, radiotherapy enhances TGF-β signaling [193]. Elevated TGF-β levels may be a result of CAF activation and may also influence tumor progression by further modulating CAF activity [194].

In addition, molecules secreted by CAFs are able to influence the outcome of radiotherapy. It has been shown that the exosome miR-196a-5p from CAFs enhances the radiation resistance of lung cancer cells by down-regulating NFKBIA [195]. CAFs produce IGF1/2, CXCL12, and β-hydroxybutyrate that induce autophagy and promote the recovery of cancer cells from radiation damage after radiotherapy [196]. CAF-derived soluble factors mediate measurable changes in undifferentiated macrophages and down-regulate pro-inflammatory features of M1-polarized macrophages [197]. It has also been shown that CAFs are radioresistant, with significant changes in oxidative metabolic indices. CAFs that survive in radiation therapy may alter the fate of the associated cancer cells [198]. IL-22 produced by CAFs is able to increase the proliferation, invasion and migration of lung cancer cells [199]. It has been shown that in lung adenocarcinoma CAF is able to induce a tolerogenic phenotype in DC cells and enhance immunosuppression. However, ionizing radiation was able to reverse the CAF-mediated immunosuppressive effects [140].

Targeted radionuclide therapy (TRT) is a type of cancer therapy, and radionuclide therapy targeting FAP is a hot topic of current research. It has been shown that in preclinical lung cancer mouse models, [^225^Ac]Ac-DOTA-4AH29 and [^131^I]I-GMIB-4AH29 exhibited high and sustained tumor targeting, which prolonged the survival of mice with aggressive tumors; furthermore, the binding of PD-L1 ICB to [^225^Ac]Ac-DOTA-4AH29 TRT enhanced its efficacy [200]. In a clinical trial that included nine patients with advanced lung cancer, ^177^Lu-FAP-2286 treatment resulted in improvements in overall health, symptom response, and quality of life [201]. In addition to this, targeting FAP is more often used as a tracer. A clinical trial of [^68^Ga]Ga-FAPI-RGD, a dual-targeted heterodimer PET tracer that recognizes both FAP and integrin αvβ3, included 51 patients with lung malignancies. The results showed that [^68^Ga]Ga-FAPI-RGD PET/CT had a higher primary tumor detection rate, higher tracer uptake, and better metastasis detection rate compared with [^18^F]FDG PET/CT [202]. Another study developed a novel radiolabeled FAP inhibitor probe [^18^F]AlF-FAPI-74 for non-invasive monitoring of the effects of anti-FAP CAR T-cell therapy in solid tumors, demonstrating its potential for detecting changes in FAP expression in the tumor microenvironment and serving as a biomarker for predicting and monitoring response to FAP-targeted therapy [203]. In addition, a novel FAP-targeted diagnostic and therapeutic integrated probe, ^68^Ga/177Lu-labeled DOTA-FAPT, exhibited high stability, hydrophilicity, and strong targeting affinity, and showed excellent tumor uptake, diagnostic efficacy, and therapeutic potential in mouse models and lung cancer patients, with a promising future for application [204]. In an international multicenter retrospective analysis, 71 patients with various types of cancers, including 9 lung cancer patients, were treated with both 18F-FDG and 68Ga-labeled FAP inhibitors. Compared with ^18^F-FDG, ^68^Ga-FAPI had a higher diagnostic performance for cancer staging and restaging in various indications [205].

In conclusion, the importance of CAFs in the tumor microenvironment and their complexity profoundly affects the efficacy of radiotherapy. Radiotherapy can mainly produce immunosuppressive effects by altering the phenotype of CAF. However, the development of radionuclide therapy targeting CAF-specific markers, such as FAP, for diagnostic and therapeutic purposes offers new hope for overcoming tumor immunosuppression and improving the efficacy of radiotherapy.

## 5. Discussion

CAFs have emerged as key regulators in the immune microenvironment of lung cancer, where they orchestrate a complex network of interactions that have a major impact on tumor progression and treatment outcome. As the “architects” of the microenvironment, CAFs exhibit multifaceted roles, including ECM remodeling and immune regulation. Their ability to recruit and polarize immune cells highlights their central role in the formation of an immunosuppressive environment conducive to tumor immune evasion. This dynamic interaction between CAFs and immune cells highlights the potential of targeting CAFs to remodel the tumor immune microenvironment and improve efficacy.

The interaction between CAFs and radiotherapy adds another layer of complexity to their role in lung cancer. While the main goal of radiotherapy is to induce tumor cell death, it also has profound effects on the tumor microenvironment. As we discussed in the previous sections, CAFs can directly regulate the response to radiotherapy by promoting the radioresistance and recovery ability of cancer cells; they can also affect the tumor TME by secreting some cytokines, resulting in a microenvironment of immunosuppression and, thus, indirectly affecting the efficacy of radiotherapy. Secondly, radiotherapy can activate CAFs, leading to further remodeling and immunosuppression of the ECM, which may ultimately limit the efficacy of radiotherapy. However, we also addressed that new evidence suggests that combining CAFs as targets with radiotherapy can enhance anti-tumor immunity [206]. Therefore, strategies aimed at inhibiting CAF-derived signaling pathways or removing CAFs have shown promise in preclinical models, paving the way for novel combination therapies.

Despite these advances, a number of challenges remain. An important aspect of CAF biology that deserves further attention is its heterogeneity. CAFs are not a uniform population of cells, but rather consist of distinct subpopulations with different origins, functions, and molecular characteristics. However, the classification of CAF subtypes currently lacks a standardized framework, as its definition is largely based on individual study analyses and methods. This variation in classification criteria stems from differences in experimental methods, such as single-cell sequencing, immunohistochemistry, or functional assays, as well as the specific markers or pathways emphasized in each study. For example, some researchers classify CAFs based on their functional roles (e.g., myCAFs, iCAFs, and apCAFs), while others focus on molecular features or spatial distribution in the TME. This lack of consensus complicates cross-study comparisons and may hinder the development of universal therapeutic strategies for CAFs. Going forward, a unified classification system that integrates the molecular, functional, and spatial characteristics of CAFs is critical to advancing our understanding of the role of CAFs in cancer progression and improving the precision of CAF-targeted therapies.

CAFs play dual roles in promoting and inhibiting tumor progression, and this functional diversity is also influenced by spatial and temporal factors in the tumor microenvironment as well as by interactions with other stromal and immune cells. The heterogeneity of CAFs poses a great challenge for targeted therapies because indiscriminate depletion or inhibition of CAFs may inadvertently disrupt their potential antitumor functions. Secondly, although we have searched as much as possible for evidence to describe the role of CAFs with various immune cells in lung cancer, we have also found directions that have not been investigated so far, such as the relationship between mast cells (MCs) and CAFs. MCs also have two sides in tumors. As cancer promoters, they produce different pro-angiogenic molecules such as VEGF-A, VEGF-B, FGF, heparin, stem cell factor (SCF), etc. [207,208,209,210]; on the other hand, MCs have anti-tumor effects. By producing mediators such as chondroitin sulfate, TNF, IL-1, and IL-6, it increases inflammatory response and induces apoptosis in tumor cells [211,212]. In pancreatic cancer, secretions from activated MCs induce tumor proliferation and TME changes, especially transforming fibroblasts into an iCAF phenotype [213]. However, there are currently no relevant studies on lung cancer. Future studies should focus on elucidating the specific molecular mechanisms by which CAF interact with immune cells and their modulation by radiotherapy. In addition, the development of biomarkers to identify subpopulations of CAF with pro- or anti-carcinogenic properties is essential for patient stratification and personalized treatment strategies.

The applications and study of TME in the clinic are very important and wide-ranging. For example, specific components in TME can be used as therapeutic targets, and biomarkers in TME can also be used for tumor diagnosis, staging, and prognostic assessment. In addition, by analyzing the heterogeneity of TME, individualized treatment plans can be developed for patients. However, there are many limitations of TME research in the clinic. For example, TME is highly heterogeneous within tumors and among different patients, which makes the development of universal treatment regimens complex and difficult. The methods for assessing TME have not yet been fully standardized, resulting in limited reproducibility and comparability of results, and dynamic changes in TME over time during treatment add to the complexity of developing treatment strategies. Existing technologies still have limitations in resolution and sensitivity, making it difficult to fully capture the complexity of TME and its specific changes. Therefore, although TME shows great potential in basic research, its clinical translation still faces many challenges. CAFs, as key components in the TME, have not been fully elucidated in terms of their functional diversity and dynamic changes in the TME, which limits our comprehensive understanding of their roles in tumor biology as well as the development of relevant therapeutic strategies. The functional status and phenotype of CAFs change dynamically in response to tumor progression, therapeutic interventions, and changes in microenvironmental signals. As we have discussed above, radiotherapy affects the altered phenotype and function of CAFs, which in turn affects the function of other cells in the TME and the formation of an immunosuppressive environment. In future studies, in addition to systematically analyzing the subpopulation composition of CAFs and their functional characteristics with the help of high-throughput technologies such as single-cell sequencing, spatial transcriptomics, in order to clarify the specific mechanism of their role in the TME, we should also pay attention to the dynamic evolution of CAFs in the course of treatment, and monitor the changes of CAFs and their impact on the response to treatment. This will provide an important basis for developing more precise treatment strategies.

In conclusion, CAFs play a central role in shaping the immune microenvironment and modulating the response to radiotherapy in lung cancer. Their multidimensional roles and dynamic interactions with immune cells and therapeutic interventions highlight their potential as therapeutic targets. By unraveling the complexity of CAF biology and developing innovative strategies to disrupt their pro-tumorigenic functions, we can pave the way for more effective combination therapies that harness the immune system and enhance the efficacy of lung cancer radiotherapy.

## Figures and Tables

**Table 1:** Markers of CAFs in lung cancer.

Marker	Function	Specific Role in Lung Cancer	Clinical Significance	Refs
α-SMA	Cell shrinkage, matrix remodeling	A core marker of CAF activation that promotes tumor invasion and fibrosis.	High expression is associated with poor prognosis	[63]
FAP	Extracellular matrix degradation, immunomodulation	Promote tumor growth and metastasis and inhibit anti-tumor immune response.	Associated with chemotherapy resistance and shortened survival	[45]
PDGFRβ	Cell proliferation, migration	High expression of CAF in lung cancer drives mesenchymal remodeling and angiogenesis.	Potential markers for targeted therapies	[64]
FSP1 (S100A4)	Cell migration, metastasis	Specifically labeling lung cancer CAF subpopulations, metabolic plasticity can drive tumor invasion and metastasis in non-small cell lung cancer cells.	Targeted metabolic reprogramming provides therapeutic window for enhanced glycolysis inhibition	[65]
Tenascin-C	Cell migration, metastasis	Significantly expressed in the microenvironment of lung cancer, promoting cancer cell invasion and metastasis.	High expression predicts advanced staging	[66]
Periostin	EMT	Periostin expression was positively correlated with the EMT markers Snail and Twist and with lung cancer stage, in which mature epithelial cells undergo phenotypic morphological changes and become invasive, motile cells.	Higher periostin levels correlate with poorer overall survival	[67]
Vimentin	Cell migration and EMT	High expression in CAFs enhances lung cancer cell metastasis by inducing EMT signaling; correlates with immunosuppression in the tumor microenvironment.	Associated with late staging and increased risk of metastasis	[45]
COL1A1	Malignant progression and metastasis	Higher proportion of COL1A1-positive CAFs associated with shorter patient survival.	Predicting TME-dependent survival expectancy and treatment benefits	[68]
COL5A1	Regulates collagen fiber assembly and matrix structure	COL5A1 promotes metastasis by regulating protease activity and migration-associated proteins, and inhibition of its expression reduces invasiveness and enhances chemosensitivity.	Potential therapeutic targets	[69]
P-glycoprotein	Reduces drug retention and increases drug outflow	Induced by the AKT/Sox2 signaling pathway, P-GP expression induced chemoresistance in NSCLC cells.	Associated with chemotherapy resistance	[70]
Adipocyte enhancer-binding protein 1 (AEBP1)	Promotes proliferation, migration, invasion and transfer	AEBP1 is not only oncogenic in epithelial tumor cells, but may also be oncogenic in stromal cells.	Overexpression is associated with the prediction of poor prognosis in patients with pulmonary SCC	[71]

## Data Availability

No new data were created or analyzed in this study.

## Abbreviations

The following abbreviations are used in this manuscript:

CAF	Cancer-associated fibroblasts
ECM	Extracellular matrix
NSCLC	Non-small cell lung cancer
SCLC	Small cell lung cancer
BM-MSC	Bone marrow-derived mesenchymal stem cells
EGFR	Epidermal growth factor receptor
ALK	Activin receptor-like kinase
ROS	Reactive oxygen species
TME	Tumor microenvironment
TIME	Tumor immune microenvironment
TGF-β	Transforming growth factor-β
α-SMA	α-smooth muscle actin
FAP	Fibroblast activation protein
VCAM-1	Vascular cell adhesion molecule-1
CAF-CM	CAF-conditioned medium
NF-CM	Normal fibroblast-conditioned medium
EMT	Epithelial-mesenchymal transition
SDF-1	stromal cell-derived factor-1
EndMT	Endothelial-mesenchymal transition
FSP1	Fibroblast-specific protein-1
HMEC-1	Human microvascular endothelial cells
ILK	Integrin-linked kinase
MRTF	Myocardin-related transcription factor
FAPIs	FAP inhibitors
CXCL12	C-X-C Motif Chemokine Ligand 12
IL-6	Interleukin-6
COL1A1	Collagen type I alpha 1 chain
PDGFR	Platelet-derived growth factor receptor
SCC	Lung squamous cell carcinoma
myCAFs	Myofibroblast-like cancer-associated fibroblasts
PDAC	Pancreatic ductal adenocarcinoma
HNSCC	Head and neck squamous cell carcinoma
LUAD	Lung adenocarcinoma
MMPs	Matrix metalloproteinases
iCAFs	Inflammatory CAFs
HGF	Hepatocyte growth factor
IMC	Imaging mass cytometry
non-NE	Non-neuroendocrine
apCAF	Antigen-presenting CAFs
mCAF	Matrix CAF
TAMs	Tumor-associated macrophages
TNF	Tumor necrosis factor
MMT	Macrophage-myofibroblast transformation
TANs	Tumor-associated neutrophils
CXCR2	C-X-C chemokine receptor 2
G-CSF	Granulocyte colony-stimulating factor
GM-CSF	Granulocyte-macrophage colony-stimulating factor
NK	Natural killer
IFN-γ	Interferon-γ
DCs	Dendritic cells
TDO2	Tryptophan 2,3-dioxygenase
MDSCs	Myeloid-derived suppressor cells
Fgl2	Fibrinogen-like protein 2
PMN-MDSC	Polymorphonuclear myeloid-derived suppressor cells
M-MDSCs	Mononuclear myeloid-derived suppressor cells
Th	Helper T
CTL	Cytotoxic T lymphocytes
Foxp3	Forkhead box P3
CFR	CD8^+^ T cell/CAF ratio
MMPs	Matrix metalloproteinases
RT	Radiotherapy
RIPF	Radiation-induced pulmonary fibrosis
HD-RT	High-dose radiotherapy
MCs	Mast cells
SCF	Stem cell factor
